# Preparation of Cefquinome Sulfate Proliposome and its Pharmacokinetics in Rabbit 

**Published:** 2013

**Authors:** Qiang FU, Hua-Lin FU, LUO Huan, Wei ZHANG, Guang SHU, Meng-Jiao LIU, Feng-Ying DENG, Jun HU

**Affiliations:** *Department of Pharmacy, College of Veterinary Medicine, Sichuan Agriculture University, Ya, an, Sichuan, 625014,China. *

**Keywords:** Cefquinome Sulfate, Proliposome, HPLC, Pharmacokinetics, Solid dispersion

## Abstract

Cefquinome Sulfate (CS) is a fourth-generation cephalosporin, which has been developed solely for veterinary use. It shows potent antibacterial activity against a broad spectrum of bacterial species. However, Cefquinome is susceptible to hydrolysis, which limiting its clinical employment efficacies to some extent. So, in this study, to increase Cefquinome Sulfate biological half-life, a novel Cefquinome Sulfate proliposome was prepared by solid dispersion and effervescent techniques and characterized for morphology, particle size, entrapment efficiency and in vitro release. A Reversed Phase-High Performance Liquid Chromatography (RP–HPLC) method was first chosen and established to determine the drug concentration in plasma after intra muscular (IM) administrating Cefquinome Sulfate solution and liposome at a single dosage of 18 mg/kg in rabbit. Then their pharmacokinetics in vivo was compared. Results showed that the received liposome was milky white suspension, spherical or ellipsoidal in shape. The mean particle size was 203±5 nm and the entrapment efficiency was 53.5±0.16%. The cefaquinom sulfate solution and liposome both followed a two compartment model, in vivo. The pharmacokinetic parameters for the solution and liposomal formulations were measured as follows: t_1/2__α_ were (1.214 ± 0.135) h and (1.395 ± 0.113) h, t_1/2__β_ were (8.752 ± 0.846) h and (16.503 ± 1.275) h, AUC*(0-24) *were (49.582 ± 9.173) (mg·h)/L and (138.727 ± 11.034) (mg·h)/L, CL/F were (0.357 ± 0.015) L/(h·kg) and (0.127 ± 0.012) L/(h·kg), MRT_(0-24)_ were (2.68 ± 0.229) h and (5.945 ± 0.479) h, respectively. It could be clearly seen that t_1/2__β_ of liposome prolonged (p < 0.05), AUC and MRT both increased remarkably (p < 0.01), CL/F decreased. Results indicated that this preparation has more residence time and exhibits some sustained–release tendency.

## Introduction

Cefquinome Sulfate (CS) is a fourth-generation cephalosporin, which has been developed solely for veterinary use. It shows potent antibacterial activity against a broad spectrum of bacterial species, such as a large number of Gram–positive bacterium, some Gram–negative bacterium, vibrios, spirochete, mycoplasma, *etc *(1). The antibiotic has been extensive use for treatment of cattle and pig against bacterial infections of respiratory tract and the udder (2). However, like other *β*-lactam compounds, CS is chemically unstable. This is due to susceptibility of *β*-lactam ring to acidic or alkaline catalyzed hydrolysis (3). In addition, the short elimination half–life, wide tissue distribution, large dosage, frequent drug administration, comparatively evident hormesis and animal’s stress reaction after intramuscular injection of commercial preparations have restricted the therapeutic use of CS (4, 5).

Liposomes are vesicles having concentric bilayers of lipids (6). Liposomes can prolong the half-life of drugs in blood and raise their therapeutic index, but the potential instability of liposomes can restrict their usefulness (7, 8). Several methods, such as freeze-drying (9, 10) and proliposome technique (11, 12) have been developed to improve the stability of liposome. We have employed a proliposome technique to develop an injectable formulation of CS that can prevent CS degradation before clinical use. Liposomal entrapment of CS was also hypothesized to enhance the biological stability of Cs upon *in-vivo *administration. This hypothesis was tested here by assessing the pharmacokinetics of CS as part of the developed formulation in rabbit following intramuscular administration making comparisons with CS solutions administered by the same route.

## Experimental

*Materials*

Cefquinome Sulfate (CS) of pharmaceutical grade obtained from institute of biomedical products in Wuhan Chang Hong (Wuhan, China). Soybean phosphatidylcholine (SPC) and cholesterol (CH) were both obtained from Chengdu Kelon Chemical Reagent Works (Chengdu, China). Methanol and acetonitrile for HPLC analysis were of chromatographic grade and come from Shanghai Ludu Chemical Reagent Works (Shanghai, China). All the other reagents were of analytical grade and used as received. Healthy rabbits, weighing approximately (2.0 ± 0.5) kg, were supplied by the Experimental Center of Sichuan Agriculture University (Ya,an, Sichuan, China). The rabbits were thoroughly examined before experimentation and were kept for 7 days to ensure their clinical conditions. They were fed with fresh green fodder thrice daily and water was provided ad libitum. The study was carried out according to the principles of Institutional Ethical committee for animal experiments.

*Preparation of the Cefquinome Sulfate proliposome (CSLS)*

A solid dispersion (13) and effervescent techniques was used to prepare CSLS (14, 15). In other words, Cefquinome Sulfate proliposome were prepared by solid dispersion technique, and then were hydrated with NaHCO_3_ solution to obtain Cefquinome Sulfate liposome by effervescent technique. The compositions of the proliposome formulation were Tween-80/SPC/CH/citric acid/ NaHCO_3_ salt at mass ratio of 6:36:18:33:7. The drug to lipid molar ratio was 1:20. In brief, 0.05 g Cefquinome Sulfate, 0.1 g Tween-80, 0.66 g SPC, 0.33 g CH, 0.6 g citric acid and 0.12 g NaHCO_3_ were dissolved in 30 ml chloroform and transferred to a round bottom flask. Then stirring at 70 rpm at 45°C in a rotary evaporator (RE–2000, Shanghai Yarong Biochemistry Pharmaceutical Factory, Shanghai, China) was continued, until the organic solvent was completely removed to form Cefquinome Sulfate solid granules and (or) powder (Cefquinome Sulfate proliposomes). The proliposomes were solidified at 4°C. 

Cefquinome Sulfate proliposomes were hydrated with NaHCO_3_ solution (5.0%, w/v) and rapidly converted into a liposome suspension under constant shaking for a period of 10 min prior to use.

*Liposome characterization*

The morphological observation was performed under an H-6010 type transmission electron microscopy (H–6010A–2 TEM , Japan Hitachi company ) after negatively staining CSLS with 2% phosphotungstic acid. The particle size and size distribution was measured by Mastersizer type 2000 laser scattering particle size analyzer (British Malvern company).

*Entrapment efficiency*

The CSLS and free drug were separated by means of refrigerated at 12000 rpm/min for 45 min at 4 ^◦^C (16). Then HPLC-external standard method was used to measure the content of free drug and total drug respectively. The percentage of EE(%) was calculated according to the following Equation:

$\mathrm{EE}\left(\%\right)=\frac{\mathrm{amount}\mathrm{of}\mathrm{total}\mathrm{drug}-\mathrm{amount}\mathrm{of}\mathrm{free}\mathrm{drug}}{\mathrm{amoun}\mathrm{of}\mathrm{total}\mathrm{drug}}\times 100\%$

*In-vitro release behavior*

Release evaluation of CS from liposome was performed with a dialysis method (17). 2 mL of liposomal suspension was embedded in a dialysis bag, the ends of which were fastened with polypropylene clamps and then placed in a flask containing 100 mL Phosphate buffer solution (PBS, pH 7.0) as the release medium. The whole set was put into a Water–bathing Constant Temperature Oscillator (HZS–H, Dong Ming Medical Treatment Pharmaceutical Factory, Harbin, China) and the temperature was thermostatically maintained at 37±1^◦^C, with an agitation speed of 100 rpm/min. At predetermined time intervals, 2 mL of the simples were withdrawn and an equivalent volume of fresh PBS was replenished immediately. After appropriately diluted and filtered, simples were assayed to detect the release amount of CS by HPLC method. Simultaneously, Cefquinome Sulfate solid (dissolved in pH7.0 PBS) treated similarly was considered as a negative control. The accumulative release percentage of CS (ARP, %) was calculated according to the following equation:

$\mathrm{ARP}=\frac{m}{M}\times 100$

Where m was the amount of CS released from liposome suspension into release medium from the beginning (0) to the scheduled time (t), and *M *was the amount of total drugs in liposome suspension. 

*HPLC analysis (* 18 *)*

*Chromatographic conditions and system suitability*

Analysis was performed using a ProStar HPLC system (LC–10A VP, Shimadzu liquid chromatograph, Kyoto, Japan) was composed of a quaternary pump (LC-20 AT), a vacuum degasser, a thermostatied autosampler, a column thermostat(CTO–10A) and a RF–10AXL UV detector. Data collection and processing were performed using CLASS–VP Ver.6.1 workstation software (Shimadzu Corporation). The separations were performed on a Kromasil C18 (250 mm×4.6 mm I.D., 5 μm particle size) reversed–phaseanalytical column (Dikma Technologies, Beijing, China), which was protected by a Shimadzu Shim–Pack guard column (C18, 10 mm × 4.6 mm). The mobile phase consisted of a mixture of 0.83% phosphoric acid (pH 2.8 ± 0.1) and acetonitrile (86:14, v/v), with a flow rate of 0.8 mL/min. The UV detection was operated at 270 nm and the column temperature was 25 ^◦^C. During the assay, 20 μL of samples were injected in duplicate into the analytical column.

*Sample preparation procedures*

After thawing spontaneously, 0.3 mL of plasma and 0.9 mL acetonitrile were vortex–mixed in a 2.5 mL glass tube for 5 min. After centrifugation at 10000 rpm/min for 10 min, 0.8 mL of the supernatant was transferred to a clean Eppendorf tube and evaporated to dryness under a gentle stream of nitrogen using a nitrogen blower at 37°C in a water bath. Then the dry residues were reconstituted in 0.4 mL mobile phase and centrifuged at 12000 rpm/min for 10 min. After filtering through cellulose acetate membranes of 0.45 μm pore diameters, 20 μL of the filtrate collected were injected into the HPLC system for analysis.

*HPLC method validation*

*The establishment of the calibration curves (in-vitro and vivo)*

The concentration of CS in all test samples (*in-vitro *and *vivo*) was analyzed simultaneously using an HPLC method. According to sample preparation procedures, stock solutions of standards were prepared in 0.3 mL of plasma with different concentrations of CS (respectively 0.25、0.5、1.0、4.0、10.0、16.0、24.0 μg/mL) to get a series of working standards that were used for the preparation of standard curve samples in plasma. And the different concentrations of CS (dissolved in pH7.0 PBS, respectively 0.25、0.5、1.0、2.0、4.0、8.0、16.0、32.0 and 64.0 μg/mL) to get a series of working standards that were used for the preparation of standard curve samples for *in-vitro *Release Behavior studies.

*Determination of Precision and Recovery*

The Precision was determined through the relative standard deviation (RSD). The precision of the assay for intra-day and inter-day determinations were evaluated by the analysis (CS in plasma) of three concentration levels (0.、4.0、16.0 μg/mL) of quality control samples (n = 5) on the same day and on three consecutive validation days. The extraction recoveries of analytes were determined by comparing the mean peak areas of the analytes in the pretreated quality control samples with those obtained from the pretreated blank plasma samples post-spiked with corresponding working solutions (n = 5). Three different concentration levels of CS in plasma were evaluated by analyzing five samples at each level.

Determination of limit of detection and quantification (19).

Limit of detection (LOD) and limit of quantification (LOQ) were calculated based on the standard deviation of the response and the slope. Calculated amounts per compound were prepared and standard mixture was injected in duplicate to verify the LOD and LOQ of each compound. Based on the reports of Armbruster (20), the detection limit was expressed as:

$\mathrm{LOD}=\frac{3.3\sigma }{S}$

Where σ is the standard deviation of the response, *S *is the slope of the calibration curve.

The quantification limit was expressed as:

$\mathrm{LOQ}=\frac{10\sigma }{S}$

*Pharmacokinetic study in rabbit*

*In-vivo *experiments were performed using 10 Healthy rabbits, weighing approximately (2.0 ± 0.5) kg, were supplied by the Experimental Center of Sichuan Agriculture University (Ya,an, Sichuan, China). According to the guidelines for Animal Experimentation, the rabbits were divided into two groups and fasted for approximately 12 h with water given . One was administrated with CSLS (5 mg/mL, equal to CS) and the other with Cefquinome Sulfate solution (dissolved in pH 7.0 PBS to obtain the same concentration) via i.m. administration at a single dosage of 18 mg/kg.

Blood samples were collected from the ear marginal vein of rabbit at 0.083、0.25、0.、1、2、4、6、8、10、12 and 24 h into plastic tubes containing 1% heparin sodium,and then centrifuged at 3000 rpm /min for 10 min to get plasma Stored it at -20°C until analysis. The calibration curves of CS was used to obtain blood drug concentration of different time.The drug concentration–time data in plasma and tissues were fitted by DAS2.0 software supplied by the Pharmacological Society of China (Beijing, China). The most appropriate pharmacokinetic model was evaluated in terms of the range of the coefficient of determination (*r*2) and comparisons of Akaike’s information criterion values (AIC).

## Results and Discussion

*Preparation of liposome and characterization*

Considering Cefquinome Sulfate crude form is chemically unstable, due to susceptibly of the carbonyl group linked to the *β*-lactam ring to suffer an acidic (H+)-or alkaline (OH-)-catalyzed attack by water molecules (21), CS was loaded into liposome by the method of solid dispersion and effervescent techniques to prepare Cefquinome Sulfate proliposome. Because of the proliposome are stored as a solid state and hydrated immediately prior to use, physical stability of liposome would be improved. In this proliposome, NaHCO_3_ solid and citric acid was a solid dispersion carrier and acid ingredient of effervescent agent, respectively. Based on effervescent dispersion principle, when these proliposome containing NaHCO_3_ solid and citric acid were hydrated with NaHCO_3_ aqueous solution was rapidly dissolved to urge lipid membrane to disperse in water. A great deal of carbon dioxide produced by the reaction of citric acid and NaHCO_3_ was released to provide an ideal situation and enough shear force to hydrate lipid membrane to form liposome.

In this study, it was found that when an appropriate amount of NaHCO_3_ solid was added to citric acid as a part of solid dispersion carrier, hydration time of the formation of liposome decreased comparison to that have no added. Furthermore, hydration time of the formation of liposome decreased with the increase of the content of NaHCO_3_ solid, which demonstrated that using appropriate amount of NaHCO_3_ solid as solid dispersion carrier could be helpful to hydrate lipids. However, the degradation of CS in alkaline environment was stronger than that of acidic environment (22). Therefore, to reduce the degradation of CS, a low amount of NaHCO_3_ solid (0.12 g) was chosen in this study.

Based on the investigation of the single factor(Rotary evaporation temperature, Amount of chloroform, The molar ratio of SPC to CH, amount of tween-80, The molar ratio of drug to lipid and the amount of citric acid and NaHCO_3_), an orthogonal experiment design (L9(4)3) were investigated to get the best preparation conditions. The results (Table 1and Table 2) showed that the molar ratio of SPC to CH, Amount of Tween-80, The molar ratio of drug to lipid and the amount of citric acid and NaHCO_3_ were the main four variables that influenced the EE. The optimization of the formulation of CS proliposome was carried out to obtain the optimal formulation composed of Tween-80/SPC/CH/citric acid/ NaHCO_3_ salt at mass ratio of 6:36:18:33:7. And, three batches of CS proliposome were prepared by using the optimal formulation to investigate reproducibility of this preparation and these results were shown in Table 3. 

**Table 1:** The factor-level of orthogonal design

**Level**	**Factor**			
	**A (SPC/CH, w/w)**	**B(Tween-80,mg)**	**C (drug/lipid, w/w)**	**D (Citric acid/**
1	2:1	100	1:10	500/100
2	1.5:1	150	1:15	600/120
3	1:1	200	1:20	700/140

**Table 2:** The results of orthogonal design

**NO.**	**A**	**B**	**C**	**D**	**EE%**
1	1	1	1	1	35.5
2	1	2	2	2	46.2
3	1	3	3	3	41.6
4	2	1	2	3	40.3
5	2	2	3	1	42
6	2	3	1	2	23.5
7	3	1	3	2	52.2
8	3	2	1	3	22
9	3	3	2	1	31
1*X*	41.1	44.67	27	36.17	
2*X*	35.26	36.73	39.17	42.63	
3*X*	37.07	32.03	47.27	34.63	
R	5.84	12.64	20.27	8	

**Table 3:** The particle size, PDI value, entrapment efficiency, and hydration time of the formation of liposome. Each value represents the arithmetic mean ± Standard deviation (SD), n = 3.

**Parameters**	**Batch**			**Average value**
	
	1	2	3	
Particle size (nm)	204 ± 6	214 ± 8	191 ± 2	203 ± 5
PDI value	0.137 ± 0.01	0.128 ± 0.02	0.131 ± 0.02	0.132 ± 0.02
Entrapment efficiency (%)	53.7 ± 0.21	52.4 ± 0.12	54.4 ± 0.16	53.5 ± 0.16
Hydration time (min)	10 ± 0.3	11 ± 0.5	9 ± 0.4	10 ± 0.4

**Figure 1:**
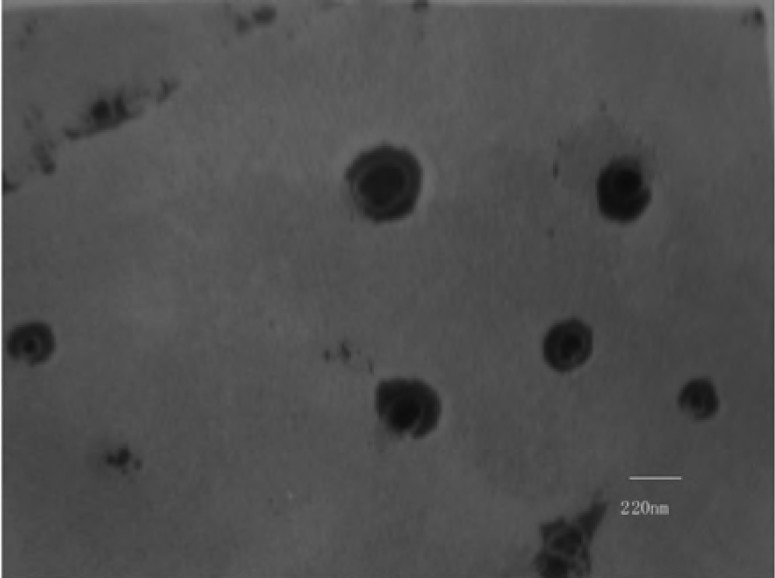
Transmission electron photograph of Cefquinome Sulfate liposome

**Figure 2:**
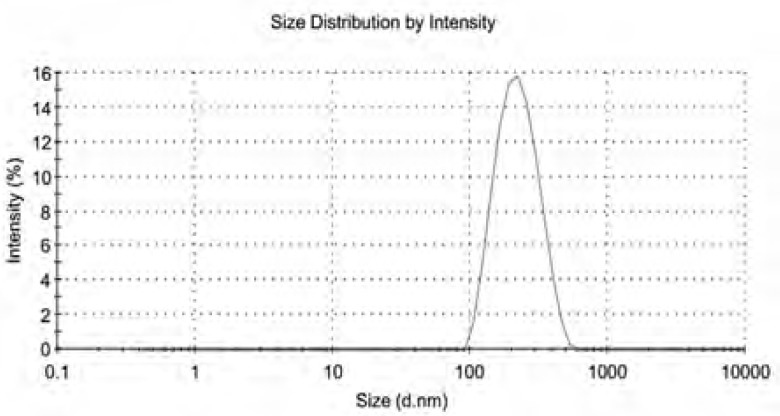
The size and distribution of Cefquinome Sulfate liposome

The CSLS prepared by the method of solid dispersion and effervescent techniques was milky white suspension. The shape observed by TEM was spherical or ellipsoidal (Figure 1). In the light of DLS detection, the particle size was 203 ± 5 nm and more than 90% of the amount was in the range of 100-1000 nm (Figure 2). PDI was found to be lower than 0.132 ± 0.02, indicating that the liposome populations were homogeneous in size. The entrapment efficiency of CSLS was 53.5 ± 0.16% with RSD of lower than 2%.

*Release studies*

**Figure 3:**
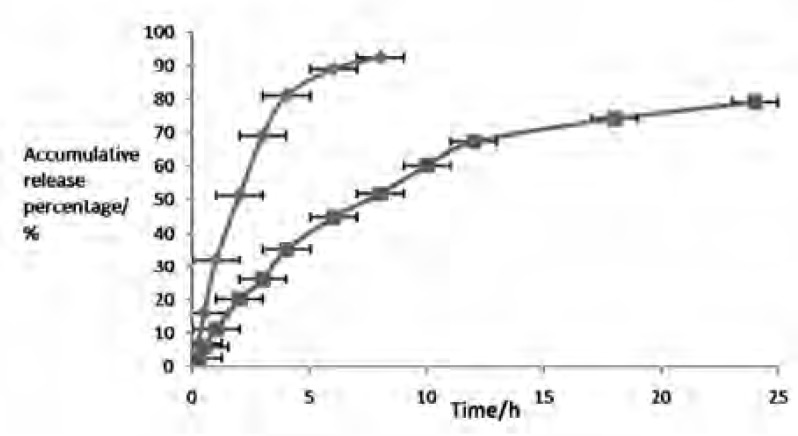
Release profiles of CS solution (◆), and CSLS (■) *in-vitro*. The values are arithmetic Mean ± Standard deviation (SD), n = 5

The release profile of an entrapped drug predicts how a delivery system might function and gives valuable insight into its *in-vivo *assimilation, distribution, metabolism, excretion, ultimately to support formulation development and preclinical studies (23). Table 4 and Figure 3 showed the *in-vitro *drug release of CS from liposome and solution. It can be distinctly seen that CS solution released much faster and ARP was 92.48% within 8 h. By contrast, CS released much slower from liposome with ARP of less than 51.78% during the same time periods. Zero and first order kinetics equation, Higuchi equation and Weibull equation were respectively utilized to analyze the release data. Results summarized in Table 5 illustrated that the release profile of CS solution could be described by First order kinetics equation, while CSLS was preferable in accordance with Weibull equation, with r of 0.9798 and 0.992 apart. Just in light of the whole information, the release of CSLS could be compartmented two stages: *i.e. *preceding rapid release and later relatively slow release, which could be explained as that drugs not encapsulated in liposome were firstly released out, accounting for the initial burst release; later, the loaded drug strode over the lipid bilayer and enter the release medium due to a concentration gradient between the medium and encapsulated drugs. Regarded as a storage system, liposome had the property of sustained–releasing the loaded drugs, as a result of prolonging the action time.

**Table 4:** The *in-vitro *release data of CS from solution and liposome

**Time (h)**	**ARP(%)** **a**	
	**Solution**	**Liposome**
0.25	6.78	2.47
0.5	15.98	5.89
1	32.01	11.08
2	51.21	20.12
3	69.11	26.08
4	81.11	35.3
6	89.07	44.72
8	92.48	51.78
10	-b	60.15
12	-	67.28
18	-	74.18
24	-	79.14

^a^: Accumulative release percentage. Expressed as [(release amount)0-t/(total amount) ×100]. b: No detection

**Table 5:** The regression equation of CS release *in-vitro*

**System**	**Model**	**Regression equation**	**r**
**Solution**	Zero order kinetics equation	ARP = 0.1109t + 0.2042	0.8431
	First order kinetics equation	Ln (1-ARP) = -0.3402t - 0.0712	0.9798
	Higuchi equation	ARP = 0.3946t1/2-0.0758	0.9547
	Weibull equation	Ln [-ln(1-ARP)] = 1.0501lnt-1.0612	0.9124
**Liposome**	Zero order kinetics equation	ARP = 0.0334t + 0.1515	0.8583
	First order kinetics equation	Ln (1-ARP) = -0.0682t - 0.1158	0.9602
	Higuchi equation	ARP = 0.1906t1/2-0.0527	0.9751
	Weibull equation	Ln [-ln(1-ARP)] = 0.9048lnt-2.2109	0.992

*HPLC method validation*

*Specificity and selectivity*

**Figure 4:**
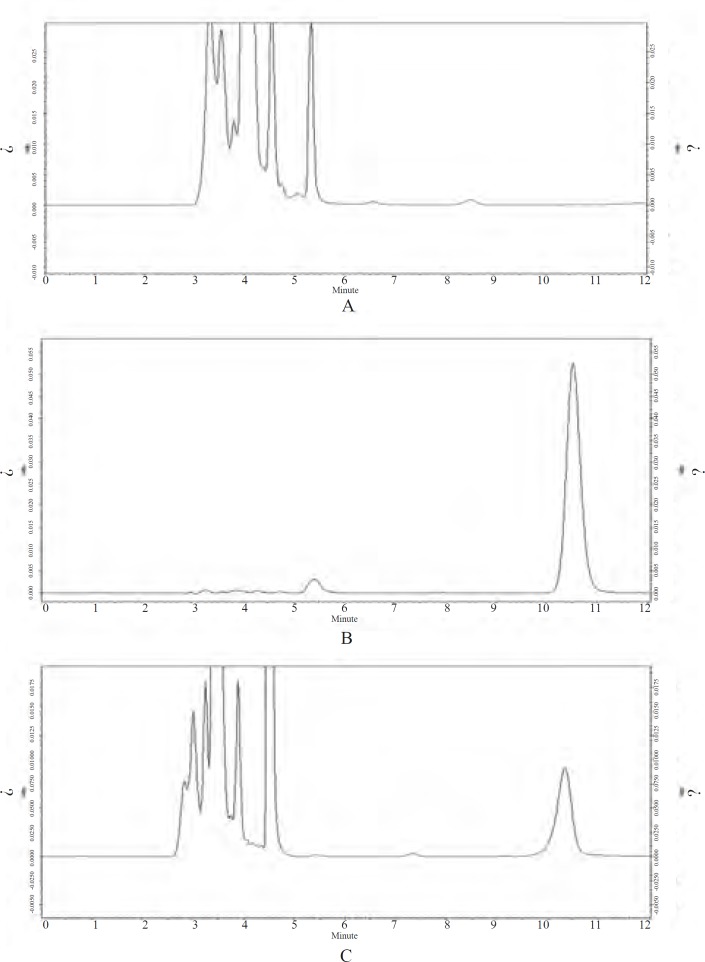
HPLC chromatogram of blank plasma, Cefquinome Sulfate (CS) and plasma simple collected from rabbit at 4 h after i.m. administration of *Cefquinome Sulfate proliposome (CSLS)*. A：Blank plasma；B：Cefquinome Sulfate (CS) (20 μg/mL)；C：Plasma simple (at 4 h).

Figure 4 represents chromatograms of blank plasma, CS solution and plasma simple collected from rabbit at 4 h after i.m. administration of *Cefquinome Sulfate proliposome (CSLS)*. No interference of endogenous peaks with blank plasma at the retention times (CS *t*R = 10.4 min). It is show that this method have strong specificity.

*The calibration curves of CS*

**Figure 5:**
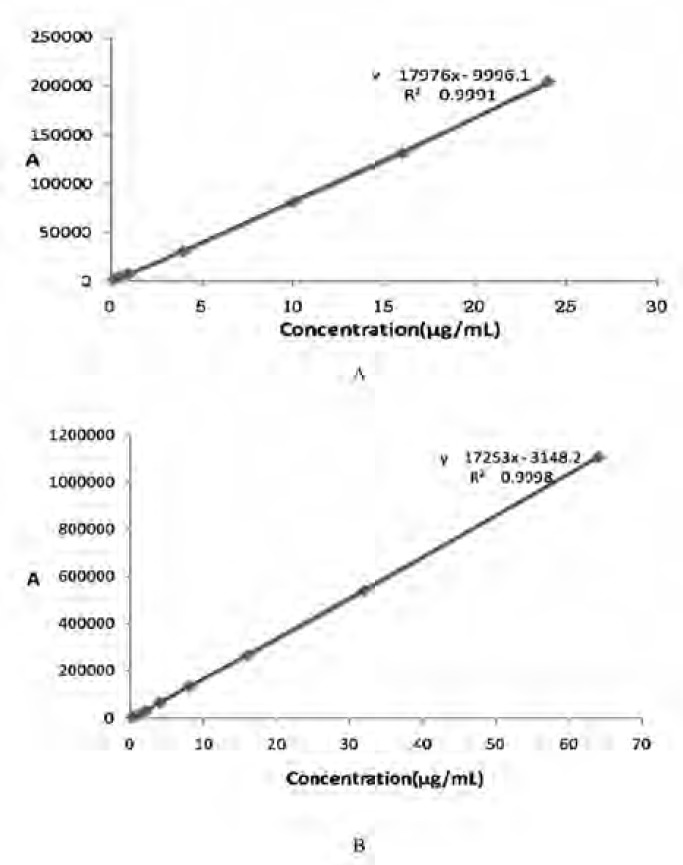
The calibration curves of CS. A: The calibration curves of CS in plasma sample. B: The calibration curves of CS (dissolved in pH7.0 PBS) *in-vitro*

According to the previously mentioned method to establish calibration curves. The results are shown in Figure 5. The linear regression equation of CS in plasma sample is *A *= 17976*C*-9996.1, with correlation coefficient *r*2 = 0.9991.The results exhibited good linear relationships between the drug concentration(*C*) and peak area (*A*) over the ranges of 0.25–24 ug/mL in plasma. The linear regression equation of CS (dissolved in pH7.0 PBS*) in-vitro *is *A *= 17253*C*-3148.2, with correlation coefficient *r*2 = 0.9998.

*Precision and recovery*

The precision for the determination of three constituents in plasma were estimated by analyzing quality control samples with low, middle and high concentrations (0.5、4.0、16.0 μg/mL). The intra-day precision (RSD) ranged from 2.99 to 3.28% and the Inter day precision (RSD) ranged from 2.02 to 5.13%. The extraction recovery was calculated by the peak area of CS in plasma samples and the same concentration of CS standards. The mean extraction recovery of CS was 84.80%、86.36% and 82.75% for low, medium and high concentrations (0.5、4.0、16.0 μg/mL), respectively, and with the relative standard deviation (RSD) for each concentration level not exceed ± 10%.

*Limit of detection and quantification*

Analysis of different concentrations of plasma samples, according to S/N = 3, the limit of detection of CS in plasma was 0.10 μg/mL and according to S/N = 10, the quantification of CS in plasma was 0.30 μg/mL. These results indicated that the method has very good Sensitivity. It is a good choice for determinating drug concentration in the plasma.

Among all analytical techniques for biological samples, HPLC method using reverse–phase column is applied the most, along with ultraviolet or visible absorbance as the detection method. In the HPLC instruments and chromatographic conditions of this study no interference of endogenous peaks with CS at the retention times in blank rabbit plasma was observed. All these experimental studies demonstrated that the established analysis method was simple, specific, accurate, reliable, prompt, sensitive and applicable for the determination of CS *in-vivo*.

*Pharmacokinetic (* 24 *,* 25 *)*

**Figure 6:**
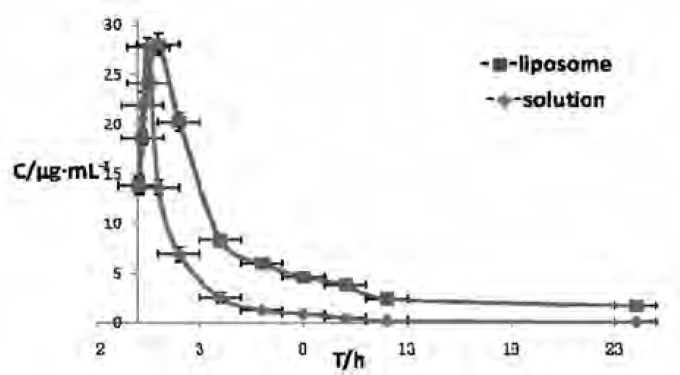
Drug concentration-time curve in rabbit plasma after i.m. Administrating Cefquinome Sulfate liposome(■) and solution( ). The symbol and vertical bar represent the mean and standard error of the mean (n = 5).

After a single i.m. administration of CSLS and CS in rabbits, the plasma drug concentration versus time profiles of the two formulations were illustrated in Figure 6. It can be clearly seen that the drug concentration in plasma rapidly reached the peak value within 0.5 h and rapidly decreased during the next 1 h, which was consistent with Liu’s study (16) that after reached the peak, a rapid clearance of the drug from the systemic circulation was observed during the next 1 h after i.m. injection of CS solution. After 1 h, the plasma drug concentration of liposome group reached the peak value and the concentration during the next 1 h was higher than that of solution group and also eliminated much slower from blood.

Based on the analysis of models and parameters (26), a two-compartment model with a weighting coefficient of 1/C2 presented the best fit to the drug concentration-time curves of the two preparations. The pharmacokinetic equation were 

C(t) = 42.066e^-0.497t^+4.537e^-0.042t^ and C(t) = 23.644e^-0.571t^ + 0.697e^-0.079t^, respectively. 

The main pharmacokinetic parameters were listed in Table 6.

**Table 6:** pharmacokinetic parameters of Cefquinome Sulfate liposome and solution in rabbits following intravenous administration

**Parameters**	**Units**	**Formulations**	
		**Solution**	**Liposome**
A	μg·mL-1	23.644 ± 6.361	42.066 ± 8.402
*α*	h-1	0.571 ± 0.045	0.497 ± 0.071
*B*	μg·mL-1	0.697 ± 0.015	4.537 ± 0.06
*β*	h-1	0.079 ± 0.008	0.042 ± 0.005
*t*1/2*α*	h	1.214 ± 0.135	1.395 ± 0.113
*t*1/2*β*	h	8.752 ± 0.846	16.503 ± 1.275*
*K*21	h-1	0.093 ± 0.007	0.097 ± 0.009
*K*12	h-1	0.084 ± 0.014	0.196 ± 0.026
*K*10	h-1	0.473 ± 0.072	0.246 ± 0.013
AUC(0-24)	mg·h·L-1	49.582 ± 9.173	138.727 ± 11.034**
AUC(0-∞)	mg·h·L-1	50.395 ± 9.270	141.262 ± 11.037**
CL/F	L·h-1·kg-1	0.357 ± 0.015	0.127 ± 0.012
*V*c	L·kg-1	0.751 **± **0.029	0.833 ± 0.027
MRT(0-24)	h	2.68 ± 0.229	5.945 ± 0.479**
MRT(0-∞)	h	3.146 ± 1.798	8.254 ± 2.571**

Note: * means significant difference between the treatments (p < 0.05), ** means highly significant difference between treatments (p < 0.01)

It can be clearly seen that the plasma drug concentration of liposome group was higher than that of solution group and also eliminated much slower from blood. The main pharmacokinetic parameters also indicated that in the plasma drug concentration of liposome group, the values of *t*_1/2__β_* ,*AUC and MRT markedly increased by about1.89-fold, 2.79-fold and 2.21-fold,respectively, in comparison to that of the solution group (p < 0.01). Furthermore, in the plasma drug concentration of liposome group, the values of CL/F and K_10_ markedly decreased to about 0.35-fold and 0.52-fold,respectively,in comparison to that of the solution group. All these results demonstrated that CS making into liposome formulation had palpable characteristics of sustained–release (27), as a result of prolonging the duration of drug concentration, reducing drug given bits and enhancing therapeutic efficiency.

When CS was Prepared to CSLS, it could overcome the limitation of quickly absorb and easy to eliminate of Conventional preparations of CS. In the groups of CSLS, the values of *t*_1/2__β_(p < 0.05) and MRT(p < 0.01) markedly increased by about 1.89-fold and 2.21-fold, respectively, in comparison to that of the solution group. In addition, CLs and K_10_ markedly decreased. All these Parameters demonstrated that CSLS gave a markedly larger MRT and longer residence time in the systemic circulation than CS solution group, exhibiting an obvious sustained–release effect. Meanwhile, in the groups of CSLS, the values of AUC markedly increased by about 2.79-fold (p < 0.01), in comparison to that of the solution group. This show the bioavailability of CSLS is obviously higher than the Solution group. Therefore, it can be concluded that liposomal CS preparation will have expansive and favorable prospects to be developed as a new formulation for Cefquinome Sulfate of high therapeutic index and sustained–release. 

## Conclusions

A novel combination of solid dispersion and effervescent techniques was developed to prepare Cefquinome sulfate liposome. The prepared liposome was milky white suspension and was spherical or ellipsoidal in shape; the mean particle size was 203 ± 5nm and more than 90% of the amount were in the range of 100-1000 nm. The proliposome exhibited good stability. An RP–HPLC method of higher specialty for the content determination of Cefquinome Sulfate liposome was first chosen and established. It was simple, accurate, specific, reliable and applicable for the analysis. Further studies will investigate the difference of pharmacokinetic parameters between Cefquinome Sulfate liposome and Cefquinome Sulfate Suspension. All these parameters and *in-vitro *release behavior studies demonstrated that CSLS exhibiting an obvious sustained–release effect. The liposomal CS preparation will have expansive and favorable prospects to be developed as a new formulation for Cefquinome Sulfate of high therapeutic index and sustained–release.
